# Implementing Remote Memory Clinics to Enhance Clinical Care During and After COVID-19

**DOI:** 10.3389/fpsyt.2020.579934

**Published:** 2020-09-18

**Authors:** Andrew P. Owens, Clive Ballard, Mazda Beigi, Chris Kalafatis, Helen Brooker, Grace Lavelle, Kolbjørn K. Brønnick, Justin Sauer, Steve Boddington, Latha Velayudhan, Dag Aarsland

**Affiliations:** ^1^ Department of Old Age Psychiatry, Institute of Psychiatry, Psychology and Neuroscience, King’s College London, London, United Kingdom; ^2^ The University of Exeter Medical School, The University of Exeter, Exeter, United Kingdom; ^3^ Psychological Medicine and Older Adults, South London & Maudsley NHS Foundation Trust, London, United Kingdom; ^4^ Ecog Pro Ltd, Bristol, United Kingdom; ^5^ SESAM—Centre for Age-Related Medicine, Stavanger University Hospital, Stavanger, Norway; ^6^ Department of Public Health, Faculty of Health Sciences, University of Stavanger, Stavanger, Norway

**Keywords:** dementia, ****cognitive impairment, telemedicine, neuropsychological assessment, geriatric psychiatry and aging, remote measurement technologies

## Abstract

Social isolation is likely to be recommended for older adults due to COVID-19, with ongoing reduced clinical contact suggested for this population. This has increased the need for remote memory clinics, we therefore review the literature, current practices and guidelines on organizing such remote memory clinics, focusing on assessment of cognition, function and other relevant measurements, proposing a novel pathway based on three levels of complexity: simple telephone or video-based interviews and testing using available tests (Level 1), digitized and validated methods based on standard pen-and-paper tests and scales (Level 2), and finally fully digitized cognitive batteries and remote measurement technologies (RMTs, Level 3). Pros and cons of these strategies are discussed. Remotely collected data negates the need for frail patients or carers to commute to clinic and offers valuable insights into progression over time, as well as treatment responses to therapeutic interventions, providing a more realistic and contextualized environment for data-collection. Notwithstanding several challenges related to internet access, computer skills, limited evidence base and regulatory and data protection issues, digital biomarkers collected remotely have significant potential for diagnosis and symptom management in older adults and we propose a framework and pathway for how technologies can be implemented to support remote memory clinics. These platforms are also well-placed for administration of digital cognitive training and other interventions. The individual, societal and public/private costs of COVID-19 are high and will continue to rise for some time but the challenges the pandemic has placed on memory services also provides an opportunity to embrace novel approaches. Remote memory clinics’ financial, logistical, clinical and practical benefits have been highlighted by COVID-19, supporting their use to not only be maintained when social distancing legislation is lifted but to be devoted extra resources and attention to fully potentiate this valuable arm of clinical assessment and care.

## Introduction

Cognitive impairment and dementia increase with age and represent major challenges for patients, their families and society. Accurate diagnosis of cognitive impairment, the degree of impairment, such as subjective cognitive decline (SCD), mild cognitive impairment (MCI) and dementia, and underlying aetiologies in older people are important tasks for the healthcare system, requiring;

- collection of history to ascertain subjective cognitive impairment
- any potential impact on function *via* activities of daily living (ADLs)
- mental status examination, including objective assessment of cognition, mood and other psychiatric symptoms that can affect cognition and provide diagnostic information
- medical/neurological examination and biomarker analyses for aetiological diagnosis (1).

The COVID-19 pandemic has heighted the need for remote offsite (i.e., virtual) cognitive assessment. Older people are at higher risk from COVID-19, due to ongoing age-related psychosocial changes, existing physical and mental health conditions and smaller social networks, on whom they may be reliant. Older adults are therefore particularly recommended to minimize risk of infection by using social distancing measures, yet the importance of a timely diagnosis of dementia remains unchanged, or has arguably increased due to the high association between COVID-19 and dementia (2). In fact, unintended consequences of such distancing may lead to reduced physical and social activity, loneliness and depression - all factors associated with more rapid cognitive and functional decline - compounding the burden on individuals and healthcare services (3). Moreover, there is also the dilemma faced by many patients regarding their concerns about potentially having dementia and wanting to speak to a clinician, offset against concerns of contracting COVID-19 should they allow a clinician into their home or visit a clinic (4). Remote memory assessments can potentially resolve this dilemma and provide an opportunity to re-evaluate how existing methods can be adapted for remote assessment and how digital technology can be used to automate cognitive assessments and data collection.

**Figure 1:**
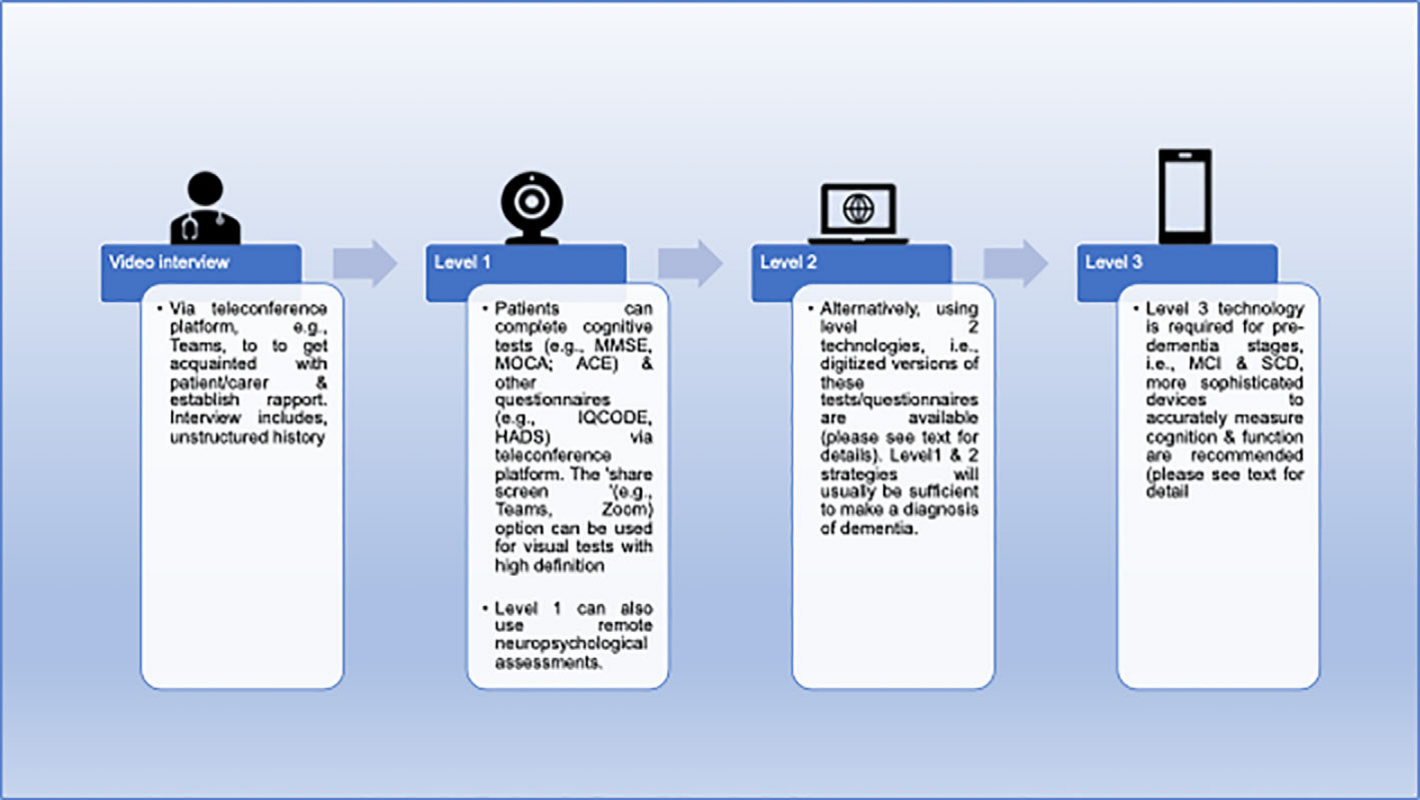
Example of remote memory clinic care pathway. ACE, Addenbrooke’s cognitive examination; HADS, Hospital Anxiety and Depression Scale, IQCODE, Informant Questionnaire on Cognitive Decline in the Elderly; MCI, mild cognitive impairment; MMSE, Mini Mental State Exam; MoCA, Montreal Cognitive Assessment; SCD, subjective cognitive decline.

Memory clinics can use a variety of approaches in this challenging situation. In the UK, regional and national guidelines have been provided (5, 6). At the simplest level, clinicians can use the telephone to interview patients and informants and ask simple questions to get an impression of mental status in addition to history. At a more systematic level, clinicians can employ structured telephone interviews [e.g. Telephone Interview for Cognitive Status (TICS) (7)] or remote versions of standardized assessment scales (e.g. eMontreal Cognitive Assessment [eMOCA (8)], telephone-Mini Mental State Examination [tMMSE (9)]. Finally, fully automated systems and related scalable digital technologies exist to measure cognition and function. **Table 1** lists potential remote memory clinic assessments and **Figure 1** provides an overview of how remote memory clinics can stratify these assessments into adapted standardized procedures (level 1), use already standardized remote instruments (level 2) or utilize automated batteries and remote measurement technologies (RMTs, level 3).

**Table 1:** An overview of how remote memory clinics can adapt standardized procedures (level 1), use already standardized remote instruments (level 2) or utilize automated batteries and remote measurement technologies (RMT, level 3).

***Domain***	**Level 1Adapting standard procedures**	**Level 2Standardized instruments**	**Level 3Automated batteries/RMTs**
***Cognition***	CDR	eMOCA MoCA Blind	Automatic Neuropsychological Assessment Metrics
	ADCS	tMMSE	CANTAB
	MoCA		Cognitive Assessment of Later Life Status
	MMSE	TAMS	Cognitive Drug Research Computerized Assessment System
			Computerized Neuropsychological Test Battery
			Mezurio
		TICS & TICSM	Mindstreams
			PROTECT
			Touch Panel-Type Dementia Assessment Scale
***Function***	ADCS-ADL	eAiADL	Altoida Medical Device
	AiADL		Residential Movement Detectors
	DAD		
	ECog		Wearable camera during ADLs
	FAQ	eMMSE	
		TICS	
***Mood***	NPI	eGAD-7	Mezurio
	PHQ		
***Motor***	UPDRS ADL section	Home video diary	GAITRite
			Gait Up
			KinetiSense
			Personal KinetiGraph

ADCS, Alzheimer’s Disease Cooperative Study; ADCS-ADL, Alzheimer’s Disease Cooperative Study-Activity of Daily Living; AiADL, Amsterdam Instrumental Activities of Daily Living Questionnaire; CANTAB, Cambridge Neuropsychological Test Automated Battery; CDR, Clinical Dementia Rating; DAD, Disability Assessment for Dementia; ECog, Everyday Cognition Scale; FAQ, Functional Activities Questionnaire; eGAD-7, electronic General Anxiety Disorder-7; MMSE, Mini Mental State Exam; MoCA, Montreal Cognitive Assessment; NPI, Neuropsychiatric Inventory; PHQ, Patient Health Questionnaire; RMDs, Residential Movement Detectors; TICS, Telephone interview for cognitive status; TICSM, Telephone interview for cognitive status modified; TAMS, Telephone assessed mental state; UPDRS ADL, Activities of Daily Living section of the Unified Parkinson’s Disease Rating Scale.

While physical examination provides information that cannot easily be captured remotely, the wealth of recent developments of digital devices and technologies represent unique opportunities for more efficient and accurate data collection, which are feasible and acceptable from the user-perspective. Remote memory clinics may also reduce the cost of face-to-face outpatient appointments, while improving the quality and relevance of cognitive monitoring and creating trial-ready cohorts for academic and commercial trials.

Active and passive digital biomarkers of cognitive domains can be collected using smartphones, tablets, personal computers (PCs), wearables and smart home sensors, virtual reality, augmented reality and even video games, that can detect changes in health status and quality of life (QoL), offering a unique opportunity to accurately and continuously track and assess changes in various physiological, motor and psychological domains. However, in terms of accuracy of measurement, acceptability and feasibility, implementation of novel strategies needs to be evidence-based and must comply with regulatory requirements, including data protection. When using adapted traditional tools, there is also some uncertainty about potentially invalidating tests by using smartphones and tablets. The device haptics usually will differ and user-interface interaction differences, as well as video and audio quality or screen size, may impact performance.

Therefore, the aim of this paper is to present a framework for virtual memory clinic assessment. To do this we will review recent practices, guidelines, scientific literature and our own experience of adapting procedures for virtual administration of diagnostic procedures relevant for memory clinics, digital cognitive assessment and RMTs for measuring function *via* ADLs, and other relevant features, such as mood and motor symptoms. Based on our experience of adapting practices for remote clinical assessments during COVID-19, the procedures for history taking, cognitive testing, functional assessment and other relevant assessments will be categorized according the three levels mentioned above:

Level 1: *ad hoc* adaptations of traditional assessments,

Level 2: specific adaptations with psychometric data available

Level 3: automated digital techniques, including RMTs.

## Methods

There have been a handful of systematic reviews of RMT-based cognitive assessment (10, 11), the last of which, to our knowledge, was published in January 2019 (12), which included data until October 2018. A summation of these reviews is included in **Table 2**, along with an update of the field since the last review. A more detailed description of this update and related emerging RMTs is given below in, ‘*Level 3: cognitive remote measurement technologies for remote memory clinics*.’ We carried out a literature review to examine new candidate RMTs for cognitive testing in remote memory clinics since November 2019 until May 2020. Google (https://www.google.co.uk/) and PubMed (https://www.ncbi.nlm.nih.gov/pubmed/) literature were searched using relevant keywords, such as, ‘digital cognitive assessment’, ‘remote digital cognitive assessment’, ‘remote cognitive assessment’, ‘self-administered cognitive test’ and ‘mobile cognitive testing’. Searches were restricted to studies published in peer‐reviewed English‐language journals and no age or sample-size restrictions were placed on articles at this stage. Other studies were identified by reviewing relevant bibliographies in original papers and reviews, as well as recent guidelines that were available to us. Conference reports were also included. The initial 732 results were examined by the lead author (AO) for duplications before the authors with experience in memory clinics (CB, CK, KB, JS, LV, DA), clinical neuropsychological testing (MB, SB) and RMT-based clinical research (AO, GL, HB) independently reviewed and reached a consensus on the final 48 eligible articles. There was a particular focus on studies involving neurotypical older adults and MCI. MCI was of interest because, during disease progression, the patient’s proficiency to carry out instrumental ADLs will be increasingly impaired, yet remote testing requires self-regulation from the patient in order to comply with testing procedures. Moreover, test logistics may overwhelm the capacity of patients inexperienced with digital technology, and those at the predementia or mild dementia stages are likely to provide better uptake of technology-use than dementia cohorts. Therefore, our focus of RMTs centered on these cohorts to ensure the results were as relevant and applicable to a timely rolling out of a remote memory clinic service as possible. Relevant and accessible journal articles that assessed the use of cognitive assessments that could be deployed offsite from the clinical setting and allowed for test data to be collected by the clinician were considered.

**Table 2:** Potential remote measurement technologies for self-administered remote cognitive assessments.

***Type of RMT***	**Examples**	**Domains tested**			**Pros**	**Cons**
***Onlineplatforms***	CANTAB (13)	Attention general memory working memory visual memory	semantic/verbal memory decision making response control	learning reaction time executive function	PCs & laptops are commonly owned by an increasing number of older adults	Limitations in hardware capabilities and internet access
	Cognitive Testing on Computer (14)	Memory processing speed	language skills visuospatial skills	constructional capabilities executive function		
	DETECT (15)	Attention selective memory	working memory information processing speed	executive function		
	GrayMatters (16)	Visual memory		executive function		
	Interactive Voice Response (17)	Declarative memory working memory	short-term memory long-term memory	semantic memory		
	MyCognition (18)	Attention psychomotor speed	working memory episodic memory	executive functioning		People who are non-fluent in English will be unable to use
	PROTECT Battery (including CogPro) (19)	Visual episodic memory spatial working memory working memory	numeric working memory verbal reasoning visual attention	task-switching delayed word recall word recognition		Questions over the generalizability of normative data, given the skewed sample favoring older people with higher levels of computer literacy
	VECP (20)	Visuospatial attention				
***Device-based***	BrainCheck Memory (21)	Immediate recall delayed recall visual attention	task switching processing speed working memory	visuospatial processing executive function	Devices & smartphones are more portable than computer-based testing.	Devices are easier to lose or damage than PCs or laptops
	Integrated Cognitive Assessment (Cognetivity)	Visual attention task switching reaction time executive functioning	working memory visual function episodic memory	semantic memory spatial memory global cognition		
	Computerized Assessment of MCI (22)	Attention processing speed verbal memory	nonverbal memory functional memory	incidental memory executive function		Visual impairments in the elderly can add to challenge when using a smaller device
	CANTAB Mobile (23)	Visual episodic memory	depression	activities of daily living		
	ClockMe System (24)	Visuospatial skills	constructional capabilities	executive function		
	Cognitive Assessment for Dementia, iPad version (25)	Recall delayed recall	semantic memory working memory	spatial orientation executive function		Non-English speakers may have limited options
	CANS-MCI (26)	Memory Language	spatial capabilities	executive function		
	CRRST (27)	Verbal memory & learning			Apps are readily available from the appropriate vendor	Handheld nature of such devices may be a challenge for those with motor or rheumatological comorbidities
	Mezurio (28)	Visuospatial memory	spatial orientation	visuospatial skills		
	Mobile Cognitive Screening (29)	Attention visual configuration language	memory orientation	calculation executive function		
	NCGG-FAT (30)	Memory Attention	processing speed visuospatial perception	executive function		
	Neurotrack Memory Health Program (30)	Visual recognition		memory		
	Spatial Delayed Recognition Span Task (31)	Visuospatial working memory				
	Touch Panel-type Dementia Assessment Scale (32)	Immediate recall delayed verbal memory	spatial orientation	spatial recognition		
***Wearables***	Cognition Kit (33)	Working memory			Can provide passive data collection or short but repeated session of active data collection compared to online platforms & smartphone or tablet-based assessment	Can be expensive
		Can be expensive			Can be collected for all people irrespective of sensory impairments or speech & language difficulties	Are easier to forget to wear/use & misplace
***Virtual reality*,** ***augmented reality & games consoles***	Altoida MD (34)	Visual attention	Orientation	spatial memory	More engaging approach to cognitive assessment & may negate any issues with poor eyesight, speech or language difficulties	Potential additional user interface complexity, additional devices & expense if compatible device must be purchased
	VSM (35)	Visual attention	task switching	executive functioning		
	Nintendo Wii balance board (36)	Spatial orientation				

CANTAB, Cambridge Neuropsychological Test Automated Battery; CANS-MCI, Computer-Administered Neuropsychological Screen for Mild Cognitive Impairment; CRRST, Cued-Recall Retrieval Speed Task; NCGG-FAT, National Centre for Geriatrics and Gerontology Functional Assessment Tool; VECP, Visual Exogenous Cuing Paradigm; VSM, Virtual Super Market.

We build these findings around a discussion of our experience of deploying remote assessments and internet-based cognitive testing in clinical practice, collection of health data and RMT assessment of function in elderly people with pre-dementia and dementia in two large ongoing multicentre studies, RADAR-AD and PROTECT and remote assessments during the COVID-19 pandemic, with a view of providing guidance into how remote memory clinics may be realized.

## Results

### Level 1: *Ad Hoc* Adaptations of Traditional Clinic Assessments

This is the simplest way of adapting to the need for remote assessments. Instruments used in pre-COVID-19 clinical settings are straightforwardly adapted for remote assessment. This has limitations if conducted telephonically as certain items cannot be performed, e.g., visual tasks in the Addenbrookes Cognitive Examination III (ACE III) and the Mini Mental State Exam (MMSE). This necessitates an adjusted score with changes in test validity. However, the benefits of this method are its ease, similarity to a standard clinical interview and simple technology and delivery. The clinician will be familiar and comfortable with this procedure, which requires no technological resources other than a telephone. Such technology is also likely to be accessible and acceptable to older populations.

The application of a standard clinical assessment *via* video call allows the use of tools already familiar to clinicians, as set out above, and can be applied in a manner more akin to the usual clinic. However, the authors experiences of such assessments *via* video is that it can take at least 50% more time. It can also be more challenging for patients due to increased cognitive load for those with attentional depletion, particularly if holding up instructions or images, although screen-sharing pre-prepared images or instructions can mitigate against this. Still, the possibility of underestimating the patients’ true cognitive level should always be considered. Mood symptoms can be easily addressed in an interview, with video providing important non-verbal information. Similarly, motor symptoms can be discussed verbally, and video offers the opportunity to observe and assess bradykinesia as well gait, stride, turning and tremor (rest and postural).

The limitations of such an approach includes the lack of standardization and evidence regarding the accuracy of paper-based tests being used in this way. In some cases, one is likely to lose sensitivity and is less likely to detect subtle changes of cognitive and functional decline, particularly in early phases of dementia. However, in cases of dementia with predominant attentional deficits, other cognitive domains may be underestimated due to the increased attentional demands inherent in the situation. Hearing or speech difficulties can add to the challenge. Hence, the clinician should evaluate the combination of sources of bias in each case.

### Level 2: Specific Adaptations With Psychometric Data Available

The instructions and content of the electronic version of the Montreal Cognitive Assessment (eMoCA) (8) are identical to the original and it is available as a downloadable app on tablets. Studies comparing the eMoCA vs MoCA are limited, as are those validating the MoCA-Blind, which has also been proposed as a suitable cognitive screening tool for telephone administration. Snowden and colleagues (8) randomly allocated participants to the eMoCA (n=182) or MoCA (n=219) from nine primary care practices. The study concluded between-group significant differences in scores (MoCA group = 26.21, eMoCA group = 24.84) and completion times (MoCA group = 10.3 min, eMoCA group = 15.3 min). However, in a recent smaller-scale study in adults (n=43) presenting with memory concerns (mean age: 72 years), the eMOCA shows convergent validity (*r*=.84) with the original MOCA, indicating the eMOCA does not significantly alter the reliability of the original scale (38).

The MoCA-Blind removes the four visual items included in the original to for patients with visual impairments. Wittich and colleagues (39) report that based on absolute score ranges, the MoCA-Blind achieves poorer sensitivity for MCI (44%) in comparison to the original MoCA (90%) but provides improved sensitivity for Alzheimer’s disease (AD) (87%), although this was still inferior to the MoCA (100%). Although the MoCA-Blind has potential for remote use, it has not been designed or validated for these purposes. All versions of the MoCA are currently free to access.

Scores of the telephone version of the MMSE (tMMSE) strongly correlate (*r*=.85) with original MMSE scores across the spectrum of neurotypical to moderately demented participants (40) and the more recent 26-item tMMSE produces scores are even closer (*r*=.88) to in-person MMSE administration in AD (9). The tMMSE involves a three-step action-based response prior to cognitive assessment, which examines memory, attention, recall, orientation and calculation (9).

The Telephone Interview for Cognitive Status (TICS) (7) was designed to examine cognitive status in AD and was proposed as an alternative to the standard MMSE (41), as both have comparable cut point scores. The TICS examines attention, short-term memory, orientation to time and place, sentence repetition, naming to verbal description, immediate recall, word opposites and praxis and has become the most commonly used telephone cognitive assessment (42). The TICS-Modified (TICSM) includes an additional delayed verbal recall component. Both TICS (*r*=.94) and TICSM (*r*=.57) scores correlate with MMSE scores (43). To date, there are several versions of the TICSM that have been developed, including those scored out of 50, 41 and 39. The latter of these versions has developed a norms calculator that corrects for age, education and sex (44). The same study found that this version of the TICSM correlated well with the MMSE (*r*=.70) and ACE-Revised (*r*=.80).

In light of recent restrictions due to COVID-19, psychometric test battery copyrighters and regulators have issued updated guidance’s to assist clinicians with resuming administration of test batteries. For example, Pearson’s (https://www.pearsonassessments.com/) have issued instructions enabling clinicians to administer their tests remotely, using screen sharing techniques for a limited time. However, this comes with the caveat that tests must not be scanned but rather projected using equipment, such as visualizers. This has allowed clinicians to more easily adapt their administration of tests to computer-based presentations using their current test batteries. The Division of Neuropsychology (DoN) (https://www.bps.org.uk/) has taken this further by providing guidance on how clinicians may be able to facilitate remote, computerized assessments in a standardized fashion.

Remote neuropsychological testing eliminates the need to adapt to online-based platforms that may be unfamiliar to services and create difficulties when needing to re-assess clients that have previously been tested using face-to-face batteries. A recent systematic review and meta-analysis has indicated that neuropsychological assessments administered through videoconferencing produce comparable validity to that of face-to-face testing (45). Remote assessments may also help clinicians access clients who are unable to attend clinics and live in hard-to-reach areas or under conditions that make them anxious at the prospect of a clinician visiting them. To this end, a home-based assessment may introduce an additional level of security and comfort for patients.

The South London and Maudsley memory service has developed a new protocol incorporating videoconferencing in order to complete neuropsychological assessments during COVID-19, involving several extensive pathways to explore the potential for testing clients remotely. All pathways involve separate electronic devices for clients and clinicians, as well as a visualizer for administration of visual tests to maintain social distancing and avoid cross contamination of material. These measures, although helpful in identifying whether remote assessments will work, do result in clinicians having to dedicate additional time to each client they wish to test. Nevertheless, remote assessments using the short form of the Wechsler Abbreviated Scale of Intelligence, Rey Complex Figure and California Verbal Learning have been administered with relative ease. Pearson’s argue that clinicians should use two cameras to observe clients during remote assessments and while this would be ideal, it is rarely possible for patients to facilitate this. This disadvantage is evident when patients perform the Rey Complex Figure, as it is not possible to observe the informative strategies that are used when completing this task. Similarly, the Hayling and Brixton test batteries have been administered remotely, with little noticeable disadvantage to patient and clinician. Feedback from clients has been relatively positive with patients feeling that they have performed as they would had the assessment been face to face.

### Level 3: Cognitive Remote Measurement Technologies for Remote Memory Clinics

The results of our literature review are listed in **Table 2** and based on our experience of remotely collecting digital biomarkers in neurotypical and cognitively impaired older adults, the authors consensus opinion was to categorize our findings into;

- online platforms
- device-based tests
- wearable RMTs
- virtual and augmented reality and games consoles.

Below we list in more detail, some of the most recent examples since the last review of the literature (12) to bring together the latest additions to the field that can be readily deployed in remote memory clinics.

#### Online Platforms

Online platforms involving cognitive tests provide a valuable means of carrying out remote cognitive assessments. As PCs pre-date tablets and smartphones, online platforms tended to be the first digital medium through which cognitive tests were digitized and modified for self-assessment. Another benefit is the popularity of PCs and laptops within many homes. However, this may bring inherent limitations in hardware capabilities and internet access, particularly in more remote areas. Also, many older adults may not be able to engage with these platforms, therefore there is a risk of only reaching those who are more able but not necessarily most representative of the general population reducing the generalisability of the normative data and the utility of the assessment tool for clinical purposes.

PROTECT (https://www.protectstudy.org.uk/) is an online longitudinal study of a healthy aging (>50 years) population funded by the National Institute of Health and Research (NIHR) for 25 years with a recruitment target of 50,000 participants (19, 46). The PROTECT cognitive test platform includes, the paired association learning task, self-ordered search, digit span task, grammatical reasoning, trail-making test B (47). There is also the option to use the CogPro system that examines immediate word recall, pattern separation stages 1 and 2, simple reaction time, digit vigilance, choice reaction time, spatial working memory, numeric working memory, delayed word recall and word recognition (48, 49). The PROTECT platform also collects data on demographic characteristics, medical history, psychiatric symptoms, lifestyle, family history of dementia, and instrumental ADLs (50). PROTECT is a versatile, modifiable and long-term platform that offers a bespoke option for remote memory clinics in the UK and is currently also adapted for use in other countries.

MyCognition (https://mycognition.com/) is a new web-based cognitive assessment tool that negates the need for specialist supervision, is designed for self-administration online *via* PC or iPad. MyCognition assesses the five cognitive domains of, attention, psychomotor speed, working and episodic memory and executive functioning using 10 short subtests and has recently been validated against the CANTAB (18), however it is worth noting that the MyCognition has not been validated in any older adults for dementia. MyCognition Quotient total scores correlated with CANTAB total scores and psychomotor speed (*r=*.604*)*, attention (*r=*.224*)* and episodic memory (*r=*.374*)* domains correlated with the corresponding CANTAB domains. However, executive function (*r=*.278*)* and working memory (*r=*.229*)* had limited divergent validity.

#### Device-Based

Device-based cognitive assessments have the benefit of being agile compared to bulkier computer-based testing. Apps can also be downloaded from the appropriate vendor onto any compatible tablets or smartphones that the patient may already own. However, the portability of such devices does mean that they are easier to misplace or damage, especially as cognitive impairment progresses, and the handheld nature of such devices, may also be a challenge in the presence of any motor symptoms.

The Integrated Cognitive Assessment (ICA, www.cognetivity.com) is a 5-min, self-administered, iPad-based, computerized cognitive assessment. It has been validated in patients with Mild-AD, MCI and multiple sclerosis and licensed as Software as Medical Device (SaMD) (51, 52). The ICA is a rapid image categorization task that measures attentional speed, accuracy and attentional speed and accuracy decay over time. It employs an artificial intelligence algorithm to improve its predictive accuracy by correlating age, gender and handedness with the composite score. The ICA does not demonstrate educational, interpretation bias or a practice effect (51) and integrates with electronic health systems. The use of the ICA aims at early detection, high-frequency monitoring of disease trajectory and response to treatment.

Mezurio [https://mezur.io] is a smartphone app that provides digital biomarkers targeting the cognitive symptoms of MCI by collecting data actively and passively *via* the patient’s smartphone with a user-friendly interface involving gamified tasks (28). Mezurio has been used in the PREVENT Dementia study and the UK Alzheimer’s Society GameChanger Study (53), with high user-compliance reported. Mezurio adapts to the user’s abilities when assessing memory (episodic, semantic, spatial memory), executive functions (attention, planning), verbal free-recall and fluency. Mezurio provides a broad spectrum of cognitive testing well-validated and easily deployable RMT in MCI cohorts.

BrainCheck Memory (https://braincheck.com/individuals/memory) is available on any Apple device and has been modified to detect age-related cognitive decline by measuring immediate and delayed recall, Trail Making Tests A and B, Stroop Test and Digit Symbol Substitution Task. In a recent large cohort study (54) in participants aged >49 years, BrainCheck Memory was administered by research staff, with scores significantly correlating with Saint Louis University Mental Status exam scores, Mini-Mental State Examination (MMSE) scores and MoCA scores. BrainCheck Memory was able to differentiate healthy controls from cognitively impaired participants (*p*=.02) and BrainCheck Memory composite scores were found to have a sensitivity of 81% and specificity of 94%.

The “Novel Assessment of Nutrition and Ageing” (NANA) touchscreen interface has been tested in 40 neurotypical elderly (mean age: 72 years) care home residents where it was deployed daily (55). Cognitive NANA data produced comparable validity and reliability to standard clinical measures, such as the MMSE, Symbol Digit Modalities Test and Digit Scan tests (55).Winterlight (https://winterlightlabs.com/) is a tablet-based cognitive assessment designed to detect cognitive impairment (56) by examining linguistic markers (57). The Mindmore (https://mindmore.com/) digitized cognitive test battery has been designed to examine global cognition, processing speed and attention, learning and memory (including working memory, executive function and language. Mindmore was recently tested in 81 healthy controls aged 21-85 years and was found to significantly correlate with traditional measures (median *r*=.53) (58).

The ‘Mobile Cognitive Screening’ (MCS) Android-based app is comprised of 33 questions over 14 tests examining the cognitive domains of executive functions, orientation, abstraction, arithmetic, memory, language, visual function and attention (29). In a sample of 23 healthy controls (mean age: 82 years) and 14 people with dementia (mean age: 73 years), MCS was able to differentiate MCI and controls participants in the cognitive domains of executive, visual, memory, attention, orientation functions (*p*=<0.05) ^8^. MCS scores also correlated (mean *r* ^2^ = .57) with MoCA scores. Although providing interesting findings, the MCS has been tested in a small sample.

#### Wearables

Wearable sensors have the advantage of providing either passive data collection or short but repeated session of active data collection compared to online platforms and smartphone or tablet-based assessment. This provides an attractive alternative to memory services, who will want to use relatively short and simple measures/platforms. However, wearables can be expensive and are easier to misplace than other digital options.

The CANTAB’s n-back task has recently been adapted as part of the Cognition Kit app to be delivered *via* the Apple watch (https://www.apple.com/uk/watch/) in 30 mild-to-moderate depression participants (aged 19-63 years) (34). Participants were required to complete the n-back three times per day, in addition to mood surveys. Adherence, defined by participants completing the n-back as least once daily, was 95% and remained consistent over the 6 weeks of data collection. Daily n-back scores correlated (*r*=0.37-0.50) with standard cognitive assessments sensitive to depression (spatial working memory, rapid visual information processing).

#### Virtual Reality, Augmented Reality, and Games Consoles

Virtual reality, augmented reality and games consoles offer a unique and potentially more engaging approach to cognitive assessment and may negate any issues with poor eyesight if a headset or television screen is used. However, with this comes more user complexity, (potentially) additional devices and expense if the patient does not already have a compatible device.

The virtual reality platform, Smart Aging Serious Game (SASG), has recently been trialed in 32 amnestic MCI (aMCI) participants (mean age: 77 years) and 107 healthy controls (mean age: 77 years) (59). The SASG had a sensitivity of 84% and specificity of 74% and was superior than the MoCA, Free and Cued Selective Reminding Test and Trail Making Test for detecting right hippocampal neurodegeneration.

The Altoida Medical Device (https://altoida.com) has received Food and Drug Administration (FDA) class II medical device qualification. It provides digital biomarkers for detection of subtle microerrors in accuracy and micromovements in latency that can help detect if MCI will progress to dementia (35). The app employs a user-friendly augmented reality interface to recreate an advanced ADL in locating a recently concealed item in the immediate environment. Voice data, hands micromovements and microerrors, gait microerrors, posture changes, eye-tracking, visuospatial navigation microerrors data streams during task performance are combined to create the user’s Neuro Motor Index (NMI). In participants aged 55-95 years, the NMI provides diagnostic accuracy of 94% in predicting cognitive worsening in amyloid positive individuals who converted to Alzheimer’s disease (AD) from MCI after 5 years (60).

Game consoles have also been employed for dual-task paradigms. For example, Leach and colleagues (37) used the Nintendo Wii balance board (https://www.nintendo.co.uk/index.html) to examine sway distance, velocity, area, centroidal frequency and frequency dispersion as a single-task condition and dual-task paradigm in 20 neurotypical elderly care home residents (mean MMSE score = 28.6; mean age 87 years) over 30 days. The dual-task paradigm comprised of combined daily word search tasks administered *via* a tablet simultaneously with use of the Wii balance board. Postural sway related to global cognitive scale scores and poorer performance on the tablet-based daily word search related to a lower cognitive status. Greater variability in sway distance and area, and less variability in centroidal sway were associated with lower scores of single-task and dual-task conditions.

Neuro-World is a set of six mobile games designed to challenge visuospatial short-term memory and selective attention (61). These games allow the player to self-administer the assessment of his/her cognitive impairment level. Game-specific performance data was collected from 12 post-stroke patients at baseline and a three-month follow-up, which were used to train supervised machine learning models to estimate the corresponding MMSE scores, and were demonstrated to have great potential to be used to evaluate the cognitive impairment level and monitor long-term change (62).

### Function

Accurately measuring function is crucial to distinguish between levels of cognitive decline (i.e., SCI, MCI and dementia) and also a key outcome in AD trials, especially at the earliest stages. Function is usually measured by self-report or caregiver reports regarding the person with dementia’s proficiency in executing basic, instrumental and advanced ADLs. Scales often neglect advanced ADLs, such as social functioning, despite social functioning, loneliness and social isolation’s contribution to dementia risk and morbidity (63–65). Indexing advanced ADLs are particularly relevant during the social distancing restrictions related to COVID-19, particularly in those more at risk of social isolation, such as the old and infirm.

Zygouris and colleagues (36) used the Virtual Super Market (VSM) to recreate an instrumental ADL for six healthy and six MCI participants (mean age: 64 years). Time of task completion was significantly longer for MCI participants and VSM scores provided a 92% classification rate for the detection of MCI. Mean VSM scores also significantly correlated with scores on the Functional Cognitive Assessment Scale, Test of Everyday Attention and Rey Osterrieth Complex Figure test.

The use of technology and devices itself has proven to be a valuable ADL for indexing functional decline in MCI, with computer-based behaviors, such as mouse clicks, typing speed and pauses corelating with cognitive scores in MCI and neurotypical users (66). Couth and colleagues identified 21 key technology behaviors sensitive to early cognitive impairment, such as text-based language use, incorrect passwords, mouse movements and difficulty opening correct items (67). Active and passive assessment of function across the full spectrum of basic, instrumental and advanced ADLs using RMTs is the primary purpose of ‘Remote Assessment of Disease and Relapse - Alzheimer’s disease’ (RADAR-AD, https://www.radar-ad.org/) to improve the assessment of functional decline in early-to-moderate AD. RADAR-AD’s main aim is the development and validation of technology-enabled, quantitative and sensitive measures of functional decline in AD and to evaluate if these new measures are more precise measures of function in a real-world environment across pre-clinical-to-moderate stages of AD compared to standard clinical rating scales. RADAR-AD’s leveraging of RMTs with real-life functional endpoints intends to improve methodologies for monitoring functional decline across the AD spectrum.

### Mood Measurement for Remote Memory Clinics

Recent evidence has demonstrated that social disconnectedness, predicts higher perceived social isolation, leading to higher depression and anxiety symptoms among older people (68). The link between mood and sleep is also been well-established (69). Therefore, the potential negative psychological impact of COVID-19 may be compounded further by widely experienced sleep alterations, including disturbances in sleep quality and quantity, which also occur with increasing age and for those with dementia (70, 71).

Patient engagement with active smartphone applications, such as those developed by Remote Assessment of Disease and Relapse (RADAR) base (72, 73) offer a solution for the remote delivery of already validated questionnaires of sleep and mood (e.g., Patient Health Questionnaire, General Anxiety Disorder-7, Pittsburgh Sleep Quality Index). This type of RMT platform has the potential to provide easily accessible information to clinicians remotely, to better inform diagnoses and clinical decision making. This concept has already been developed among people with Major Depressive Disorder (MDD) to explore if longitudinal tracking using RMT can capture information predictive of depressive relapse and other key clinical outcomes (73). RMTs also offer a unique capability to provide continuous objective data, through passive data streaming methods (72). Sleep quality and quantity variables (e.g., duration of sleep and time spent in REM cycles) can be monitored remotely through actigraphy and consumer-wearable activity trackers (74, 75).

### Motor Measurement for Remote Memory Clinics

Continuous day-to-day use of wearables are an ideal medium to collect large, well-powered data on motor symptoms, either by passive use of on-body sensors or “little but often” RMT-based active protocols. Wearable sensors for the detection of motor symptoms, such as The Personal KinetiGraph (PKG), have FDA approval and have been deployed and validated in clinical trials (76). Smartwatch-based sensors have been used predominantly in Parkinson’s disease (PD) to discriminate essential tremor from postural tremor (77). Other motor fluctuations, such as bradykinesia have been remotely assessed using wearable shoe sensors and watch-like sensors to measure gait patterns (78) and dyskinesia has been analyzed *via* home video recording (79) or using home diaries (80) for some time. Wearable gyroscopes and accelerometer sensors can passively collect data during standardized motor tasks, voluntary movements and ADLs to measure dyskinesia (78), for example, KinetiSense (https://kinetisense.com) wearable triaxial accelerometers and gyroscopes and have found that dyskinesia scores collected from KinetiSense highly correlated with clinician scores (*r*=.86) (81). The GAITRite (https://www.gaitrite.com) system has been employed to examine gait in aMCI (n=15), non-amnestic MCI (n=21) comparative to healthy controls HCs (n=21) to delineate that aMCI had greater gait variability than clinical and healthy controls (82).

### Virtual Pathway for Memory Assessment

**Figure 2:**
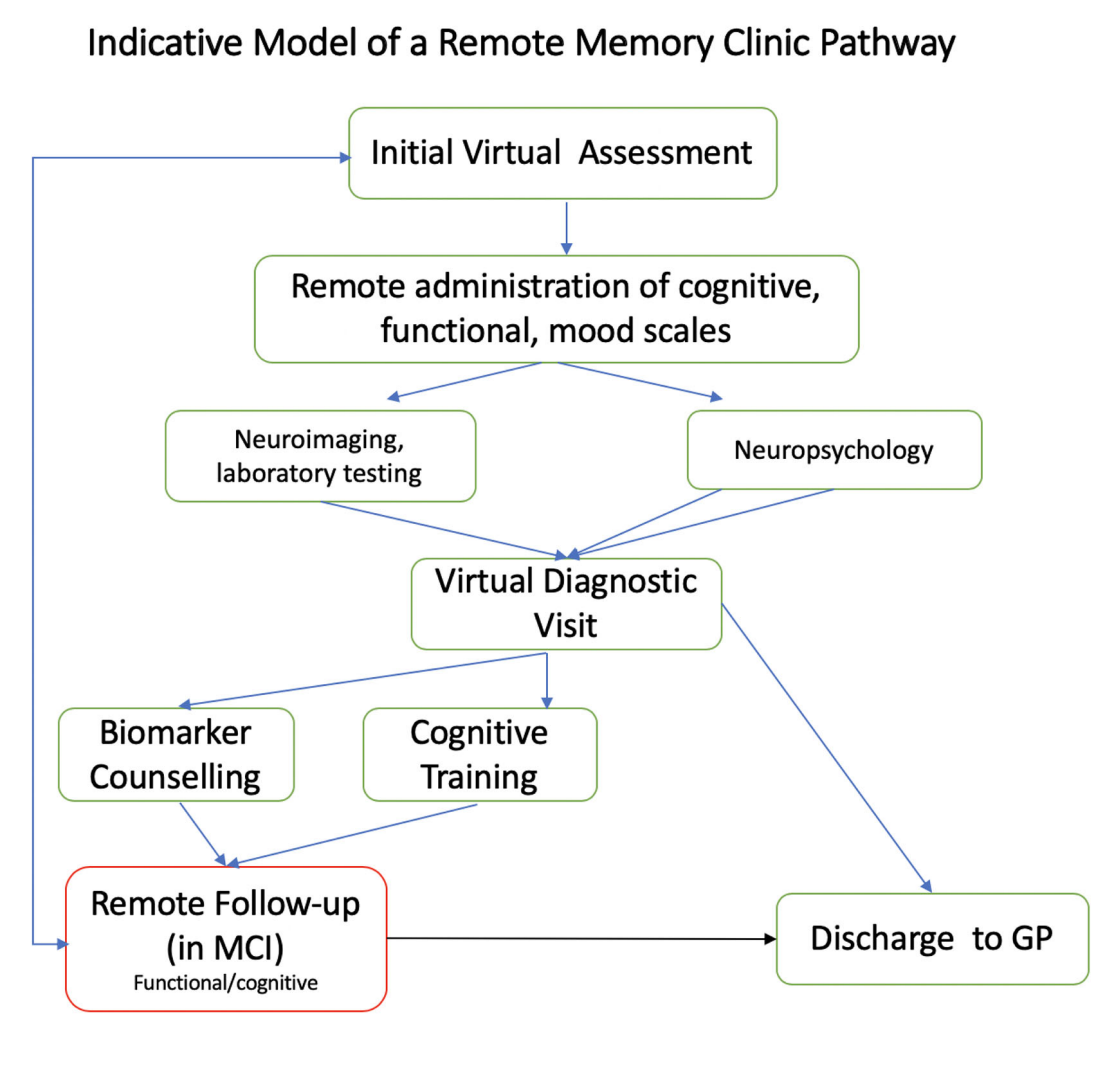
Potential clinical pathway for remote memory clinics. GP, general practitioner; MCI, mild cognitive impairment.

Outpatient-based remote memory clinics can carry-out further specialist diagnostic investigations to support accurate and timely diagnosis. Patients at risk of dementia can be followed up both remotely and in-clinic, while patients without evidence of a neurodegenerative disease (e.g., dementia biomarker-negative MCI) can be discharged to Primary Care. Patients diagnosed with prodromal dementia can be given the option of remote cognitive and functional assessments, even as part of a research framework, with these patients expected to have an annual face-to-face follow up in-clinic or until transition to clinical dementia (see **Figure 2** for potential pathway). There is also increasing evidence the computerized cognitive training can have positive effects, and these may easily by administered from online testing platforms (47, 83).

## Discussion

The COVID-19 pandemic has created major challenges for elderly people with cognitive impairment, as well as for memory clinics tasked with assessing and caring for this group. While the health systems in many countries are slowly moving back to normal, elderly people will still want to, or be expected, to reduce traveling and visiting hospitals for non-urgent causes, and in many countries infection rates are still increasing. Memory clinics therefore must adapt to this new situation and explore and offer new models and pathways for assessment and care. This situation also represents an opportunity to critically assess practices and to explore the many new technologies and methods available to assist clinicians in providing accurate, safe, and user-friendly ways of diagnosing elderly people with cognitive impairment. We have reviewed the literature and other sources, as well as reporting our own experience of deploying remote memory clinics and propose a new pathway that can be implemented immediately in memory clinics, at various levels of complexity.

As the simplest approach, Level 1 involves standard procedures, tests and questionnaires that can be administered by telephone, or, better, using available video-based platforms. While simple and requiring only a telephone, the limitations include that the psychometric characteristics may not immediately translate to this form of administration. At Level 2, we present several standardized measurements and instruments that have been digitized and have provided at least some degree of psychometric validity and reliability. Interestingly, many exciting new technologies are available to test not only cognition, but also mood and motor symptoms as well as daily functioning (Level 3). We believe that the recent challenges offer an opportunity to embrace new technology, devices, and wearables to accurately diagnose age-related cognitive disorders.

Digital biomarkers collected in remote memory clinics have significant potential for diagnosis and symptom management in older adults during and after COVID-19. Information is collected by RMTs in real-time, at a high frequency level and can also be delivered cost-effectively at a large scale. The collation of both active and passive RMT data in tandem, provides a more enriched clinical picture, while also providing a background of explanatory variables. Reduced participant burden and increased participant engagement are also among the potential benefits. Additionally, the frequency of data collected is incomparable to the momentary data capture currently employed in clinical settings. Such approaches provide accurate and continuous tracking of disease progression. These technologies may also be used to examine if some groups are more responsive than others to treatments. Such methodologies can be easily scaled-up to reach larger populations, including potentially primary care and will have relevance for future pandemics. Therefore, the scope of virtual memory clinics has significant potential to enhance current standards and should remain common practice after COVID-19.

The technologies discussed are particularly well-suited to measure and track cognitive and function and are thus excellent tools for identifying and staging cognitive impairment (i.e., SCD, MCI or dementia). However, an etiological diagnosis, i.e., identifying the disease causing the cognitive impairment, requires additional information. Although remote assessment of mood and motor symptoms, as well as the clinical history, can provide important information, biomarkers such as neuroimaging, cerebrospinal and blood markers and electroencephalogram (EEG) should be available.

Some of these biomarkers can be acquired remotely, such as EEG, sleep monitoring, and collection of saliva, urine and stools for microbiome and other analyses. For example, for the early differentiation of dementia with Lewy bodies (DLB) from AD, RMTs can enrich assessment of neuropsychiatric and dysautonomic symptoms typical in DLB (84) by capturing novel neurophysiological markers of fluctuating cognition (FC), visual hallucinations (VH), apathy or autonomic nervous system (ANS) impairments. By remotely measuring ANS function, RMTs can equip patients with person-specific protocols that complement their daily routines and lifestyle, in addition to integrating their clinical and psychosocial profiles to passively and actively collect objective contextualized data in day-to-day life over numerous timepoints. RMT-based EGG, such as Bytflies (https://www.byteflies.com), has begun to be used in epilepsy (85) and provides well-powered and contextualized data that we are using to remotely examine low-frequency spectral power in DLB (86), as longer EEG recordings in real-world settings will provide more sensitive signatures of brain changes and are more likely to capture acute episodes of FC or VH than lab-based EEG. We are also using RMTs to passively collect remote data on cardiovascular (e.g., orthostatic hypotension, postprandial hypotension) and thermoregulatory (e.g., anhidrosis, compensatory hyperhidrosis) ANS function in potential DLB cases to unmask any dysautonomia indicative of alpha-synucleinopathy. However, these biomarkers have not yet been established as diagnostic markers, thus, collection of diagnostic structural and functional neuroimaging and cerebrospinal fluid markers still requires attending a clinic.

We have provided an update on the landscape of RMT-based cognitive assessments that can be employed with immediate effect due to the urgent need to continue to deliver comprehensive memory clinic care and assessment during COVID-19, as well as a potential pathway for virtual memory assessment. Platforms, such as CANTAB and PROTECT Cognitive Test Battery offer validated and longitudinal follow-up in addition to agile design that allows for the addition of relevant tests. Other platforms, such as Neurotrack Memory Health Program (MHP, https://neurotrack.com/) combines interventions related to physical activity, diet, sleep, stress, social interaction and cognitive engagement but before any of these interventions can be used by the participant, they are required to carry out a visual paired comparison task that includes eye-tracking to provide a baseline sore of visual recognition memory. Neurotrack MHP has recently been validated in a feasibility investigation utilizing a quasi-experimental, single-arm, nonrandomized, longitudinal design in 242 healthy controls aged >51 years (31). MHP is more geared toward overarching health than cognitive testing, underlining how these online batteries can easily adapt interventions, such as cognitive behavioral therapy (CBT). Device-based cognitive assessments, such as Cognetivity and Mezurio are downloadable apps that are particularly targeted to detecting and tracking cognitive impairment. Altoida’s gamified augmented reality tasks on tablet or smartphone provides meaningful clinically relevant data and its use in largescale dementia trials makes it an ideal candidate RMT if the patient has access to the requisite hardware. The TICS has been well-validated and tested in the clinical environment (including in our clinics during the COVID-19 pandemic), producing strong construct validity compared to typical pen and paper and neuropsychological tests, aiding diagnosis while remaining a very cost-effective alternative to RMT-based assessments.

Ultimately the main argument for digital transformation in the memory services is being made for us due to COVID-19. Translating conventional pen-and-paper testing has accuracy and acceptability limitations and we believe this paper shows digital biomarkers are currently available and ready for use to this end. However, this will only be accessible for some and a key issue for memory clinics is providing a protocol and complete testing logistics chain involving caregivers or other proximal agents that can be applied to all patients. Long-term monitoring of people with MCI to identify progression to dementia is expensive and implementation of remote memory clinic pathways can provide a cost-efficient way of achieving this. Remote memory clinics can also improve research practices due to the integration of digital data onto electronic patient records that will improve data curation and availability.

A variety of computerized cognitive training interventions are available and there is increasing evidence supporting their efficacy, showing mild to moderate effect sizes in several cognitive domains in older people with MCI and dementia (83). Interestingly, several of the platforms and batteries for digital cognitive testing also offer interventions on the same platform, for example PROTECT (and its inbuilt cognitive test batteries) and MyCognition, which can often be directly tailored to the level of cognitive impairment (47). Given the lack of drug treatments for people with MCI, this is a particularly relevant feature for this group.

Although we argue the case for remote memory clinics, it is important not to neglect patients and carers who are unable to use technology for remote assessment or videoconferencing or have relevant disabilities, such as vision, speech or hearing difficulties, or other healthcare barriers related to race, economic status, disability and location. This also implies that only the more able members of the older adult community will access clinical assessment through this approach. However, arguments against digital solutions are often embedded in stereotyped views about tablet and computer use by older individuals, and there is evidence that the number of older people on line is growing fast and might even increase during COVID-19 (87). There are also limitations to rapport building and risk management should vulnerable clients become distressed during the assessment process. Clinicians should also be advised that facilitating remote testing should involve an additional pre-assessment screen to test suitability of video conferencing that factors in additional time requirements. Neuropsychological test batteries are designed and validated based on a strict set of instructions and protocols, meaning any adaptations or irregularity test administration risks invalidation. This has two major implications for clinicians. Firstly, invalidation may implicate licenses obtained through copyrighters and thus place clinicians in breach of signed agreements. Secondly, changes in administration may invalidate the norms on which scores and interpretations are based. Clinicians must therefore pay careful consideration to the implications of any adaptations for remote assessments as a result of these risks. The DoN caution that although research suggests some neuropsychological test batteries may have good reliability when administered remotely, there are still many measures that have not been assessed under these conditions, meaning the interpretation of such results must be conservative. Consideration must also be given to the risk of test material entering the public domain through remote assessments, thus undermining the validity of the tests themselves. Again, the DoN advised that clinicians must exercise caution when choosing to administer tests remotely and implement procedures that limit the risk of material entering the public domain. A further limitation to services committing to remote assessments is the publishers of tests have given notice that clinicians will need to complete training in order to qualify as registered administrators and the uncertainty regarding how long test manufacturers and licensors intend on allowing clinicians to administer their material remotely. The removal of any permission to share visual material on a computerized device would seriously hinder the potential use of visual tests. This means that, as well as a need for more research, testing the validity of remote assessments, greater flexibility on the part of test manufacturers will also be required. For example, during the COVID-19 pandemic, copyrighters have offered flexibility of how their tests can be administered, allowing the use of visualizers to share images of their tests during video calls for a limited time. It would be very beneficial for the sustainability of remote memory clinics to make such temporary permissions more permanent to allow for the development of more viable remote testing protocols. Patients’ lack of experience with RMTs and cognitive impairment present specific challenges, meaning remote memory clinics must be pragmatic (including relevant training for clinicians) and adhere to validated measures. Another consideration proving to be problematic in our experience is working with interpreters. This is already a challenge and will need separate and stratified approaches for both RMT and telephone consultations, as will sensory impairments, data protection, regulatory and feasibility issues. But the many challenges the COVID-19 pandemic has placed on memory services also provides an excellent opportunity to embrace novel technologies and approaches, both for cognitive testing and the tracking of functional status.

### Future Implications and Needs

Several platforms and devices show good measurement accuracy in small groups, future research should include confirmatory studies demonstrating diagnostic accuracy in pre-dementia diagnosis in multicentre studies with large and diverse cohorts representative of the general clinic population, as well as sensitivity to change and utility in clinical trials. In addition, comprehensive assessments, including feasibility and acceptability involving user groups, cost-efficacy studies, and ensuring adherence to regulatory requirements are required to enable evidence-based selections and priorities of devices and platforms to be used for virtual memory clinic assessments. The Horizon2020/IMI2-supported RADAR projects (https://www.radar-ad.org, https://www.radar-cns.org/) are good examples for how to achieve this.

## Conclusions

The individual, societal and public/private costs of COVID-19 are high and will continue to rise for some time but the many challenges COVID-19 has placed on memory services also provides an excellent opportunity to embrace novel technologies and approaches. A large number of possible solutions and technologies are available at different levels of sophistication. Remote memory clinics can be cost-effective and can enhance clinical assessment in the old and frail even during current or future social distancing measures. The financial, logistical, clinical and practical benefits of remote memory clinics have therefore been highlighted by COVID-19, supporting their use to not only be maintained when social distancing legislation is lifted but should be devoted extra resources and attention to fully potentiate this valuable arm of clinical assessment and care.

## Data Availability Statement

The original contributions presented in the study are included in the article/supplementary material; further inquiries can be directed to the corresponding author.

## Author Contributions

AO and DA: primary authors. CB, CK, HB, GL, KB, JS, SB, and LV: revisions and scientific content of MS. All authors contributed to the article and approved the submitted version.

## Funding

This paper represents independent research partly funded by the National Institute for Health Research (NIHR) Biomedical Research Centre at South London and Maudsley NHS Foundation Trust and King’s College London.

## Disclaimer

The views expressed are those of the authors and not necessarily those of the NHS, the NIHR, or the Department of Health and Social Care.

## Conflict of Interest

DA has received research support and/or honoraria from Astra-Zeneca, H. Lundbeck, Novartis Pharmaceuticals, Biogen, and GE Health, and served as paid consultant for H. Lundbeck, Eisai, Heptares, and Mentis Cura. CB reports grants and personal fees from Acadia pharmaceutical company, grants and personal fees from Lundbeck, personal fees from Roche, personal fees from Otsuka, personal fees from Biogen, personal fees from Eli Lilly, personal fees from Novo Nordisk, personal fees from AARP, grants and personal fees from Synexus, and personal fees from Exciva, outside the submitted work. CK is the Chief Medical Officer of Cognetivity Ltd.

Author HB was employed by company Ecog Pro Ltd.

The remaining authors declare that the research was conducted in the absence of any commercial or financial relationships that could be construed as a potential conflict of interest.
